# The effect of interfractional variation on delivered dose with ultrahypofractionated pencil beam scanning proton therapy for localized prostate cancer

**DOI:** 10.1002/acm2.70223

**Published:** 2025-09-14

**Authors:** Keyur D. Shah, Duncan H. Bohannon, Sagar A. Patel, Chih‐Wei Chang, Vishal R. Dhere, Yinan Wang, Anees Dhabaan, Hania Al‐Hallaq, Xiaofeng Yang, Jun Zhou

**Affiliations:** ^1^ Department of Radiation Oncology and Winship Cancer Institute Emory University Atlanta Georgia USA

**Keywords:** interfraction variation, prostate cancer, proton therapy

## Abstract

**Purpose/objectives:**

Proton stereotactic body radiotherapy (SBRT) using the pencil beam scanning (PBS) technique is increasingly used for localized prostate cancer (PCa) due to its potential for superior normal tissue sparing. However, interfractional anatomical variations pose challenges for accurate dose delivery, especially in ultrahypofractionation. This study investigates the dosimetric impact of these variations in PCa patients treated with PBS‐SBRT.

**Methods:**

Forty‐two low‐ or intermediate‐risk PCa patients treated with PBS‐SBRT (36.25/ 40 Gy in 5 fractions) were included. Delivered doses were calculated by applying the initial plans to HU and artifact‐corrected pre‐treatment cone‐beam computed tomography (CBCT) images. Dosimetric parameters were evaluated per fraction. Key anatomical features included clinical target volume (CTV) shifts in the superior‐inferior (SI) and anterior‐posterior (AP) directions, and volume changes in the bladder and rectum. These features were used to predict deviations in CTV D_99_ and D_0.03cc_, as well as maximum bladder and rectum doses. Univariate and multivariate logistic regression models were developed to identify significant anatomical predictors of dosimetric deviations, with odds ratios (OR) and area under the curve (AUC) values reported for predictive performance evaluation.

**Results:**

CTV AP shifts and bladder/rectum volume changes were significant predictors of dosimetric deviations. Univariate analyses indicated that bladder volume increases > 109.80 cc were strongly associated with deviations in CTV D_0.03cc_ (mean deviation = 0.33 Gy, OR = 5.78, *p* = 0.03). Rectum volume changes > 9.22 cc were the strongest predictor of rectum D_0.03cc_ deviations (mean deviation = 0.68 Gy, OR = 4.23, *p* = 0.01). AP shifts > −0.27 cm were also predictive of CTV D_0.03cc_ deviations (OR = 0.35, *p* = 0.01). Multivariate model predicting rectum D_0.03cc_ achieved the highest AUC (0.73), followed by CTV D_0.03cc_ (AUC = 0.68).

**Conclusion:**

Multivariate models incorporating bladder volume changes and CTV shifts accurately predict dosimetric deviations in PBS‐SBRT. These findings highlight the need for adaptive strategies during PBS‐SBRT to mitigate the impact of interfractional variations, optimize dose delivery precision, and reduce toxicity in high‐risk patients.

## 1 INTRODUCTION

Localized prostate cancer (PCa) remains one of the most prevalent cancers,^1^ particularly in older men, with a significant portion of cases classified as low‐ or intermediate‐risk.^2^ Traditional radiotherapy approaches for PCa have utilized long‐course, fractionated external beam radiotherapy (EBRT), typically delivered over several weeks. However, stereotactic body radiotherapy (SBRT) has emerged as a promising alternative, offering a substantial reduction in treatment duration by delivering highly conformal radiation doses over a shorter treatment course, typically five fractions or fewer.^3^ SBRT has been shown to achieve high local control rates with acceptable toxicity to surrounding organs at risk (OARs) such as the bladder and rectum.^4^, ^5^, ^6^ The biological effectiveness of SBRT is further supported by the low α/β ratio of the prostate (∼1.5 Gy),^7^, ^8^, ^9^ which makes it particularly suited to hypofractionation, allowing for higher doses per fraction to maximize tumor control.^10^, ^11^, ^12^ Several studies have shown that the therapeutic gain from hypofractionation was enhanced for tumors with a low α/β, reinforcing the utility of this approach in PCa treatment.^13^, ^14^, ^15^, ^16^

Proton therapy, and in particular pencil beam scanning (PBS), represents a valuable treatment modality for PCa due to the unique physical properties of protons, which allow for precise dose deposition at a specific depth (the Bragg peak) with minimal exit dose^17^, ^18^ and opportunities for dose escalation, such as boosting dominant intraprostatic lesions.^19^, ^20^, ^21^ PBS, an advanced proton therapy delivery technique, provides superior dose conformity and is particularly suited to SBRT, where steep dose gradients are required to spare healthy tissues from high‐dose exposure.^22^, ^23^ Studies, such as Kole et al. have demonstrated that proton SBRT offers improved dosimetric outcomes compared to photon SBRT, particularly in reducing doses to organs such as the penile bulb and urethra, without compromising target coverage.^24^ Proton‐based SBRT has become increasingly utilized in modern facilities due to its ability to reduce radiation exposure to healthy tissues and the convenience of a shorter treatment course. However, PBS is highly sensitive to interfractional anatomical variations, such as changes in bladder filling, rectal distension, and prostate motion, which can compromise the accuracy of dose delivery, particularly in ultrahypofractionated regimens.^25^

Interfractional variations are of particular concern in ultrahypofractionated treatments, where the high doses delivered per fraction leave little room for error.^26^ Daily anatomical changes during treatment can lead to deviations from the planned dose, resulting in underdosing of the clinical target volume (CTV) or overdosing of OARs, which may impact both treatment efficacy and toxicity outcomes. Although photon‐based SBRT has been extensively studied,^27^, ^28^, ^29^ there remains a lack of data specifically addressing the effects of interfractional variation in PBS‐SBRT for localized PCa, largely because many institutions are not performing PBS‐SBRT due to hardware limitations, such as the lack of cone‐beam computed tomography (CBCT).^21^ This gap highlights the need for further investigation into how these variations affect dose distribution.

In this study, we aimed to determine the thresholds for anatomical changes and assess their ability to predict dosimetric deviations. We evaluated key dose metrics, including CTV D_99_, CTV D_0.03cc_, and OAR doses for the bladder and rectum. Using logistic regression, we calculated the predictive ability of anatomical shifts and volume changes for these dosimetric outcomes. Additionally, we performed multivariate logistic regression to explore the combined effects of multiple anatomical factors on dosimetric changes. This analysis provides insight into the robustness of PBS‐SBRT under real‐world conditions and could guide future adaptive strategies for mitigating the impact of interfractional variations.

## 2 METHODS

This retrospective study was conducted on a cohort of all patients treated with PBS‐SBRT for localized low‐ and intermediate‐risk PCa at our institution between 2020 and 2022. Eligibility criteria included histologically confirmed adenocarcinoma of the prostate, a low‐ to intermediate‐risk profile (Gleason score 6–7, PSA < 20 ng/mL, clinical stage T1c–T2b), and no prior pelvic radiation. Exclusion criteria included high‐risk disease or any condition that contraindicated the use of fiducial markers or PBS.

The study protocol was approved by our institutional review board (IRB). The aim was to evaluate the differences between planned and delivered doses by accounting for interfractional anatomical variations through daily imaging and robust dose calculation. A total of 210 fractions were analyzed, with each patient receiving five fractions.

### 2.1 Patient preparation

Fiducial markers were placed in the prostate gland under ultrasound guidance at least 1 day prior to simulation. These markers were used to facilitate accurate daily localization of the prostate during treatment. Rectal spacer (SpaceOAR Hydrogel, Boston Scientific Co., MA, US) was inserted into the perirectal space for all patients to increase the separation between the prostate and rectum, minimizing rectal dose exposure. A rectal enema was used prior to simulation and each treatment fraction to ensure an empty rectum, enhancing dose delivery consistency.

Patients were immobilized in the supine position using a customized pelvic immobilization device. All patients were instructed to drink 500 mL of water approximately 1 h before both the simulation and each treatment to ensure a full bladder, thus standardizing patient setup. Computed tomography (CT) simulation scans were obtained with a 1.5 mm slice thickness for treatment planning. Immediately following CT simulation, magnetic resonance imaging (MRI) scans were acquired to aid in prostate delineation.

### 2.2 Treatment planning

Treatment plans were generated using RayStation v10B (RaySearch Lab. Stockholm, Sweden), with dose calculations performed using the Monte Carlo algorithm, ensuring high accuracy in proton range and dose distribution. Robust optimization was used to account for both setup uncertainties and range uncertainties.

**FIGURE 1:**
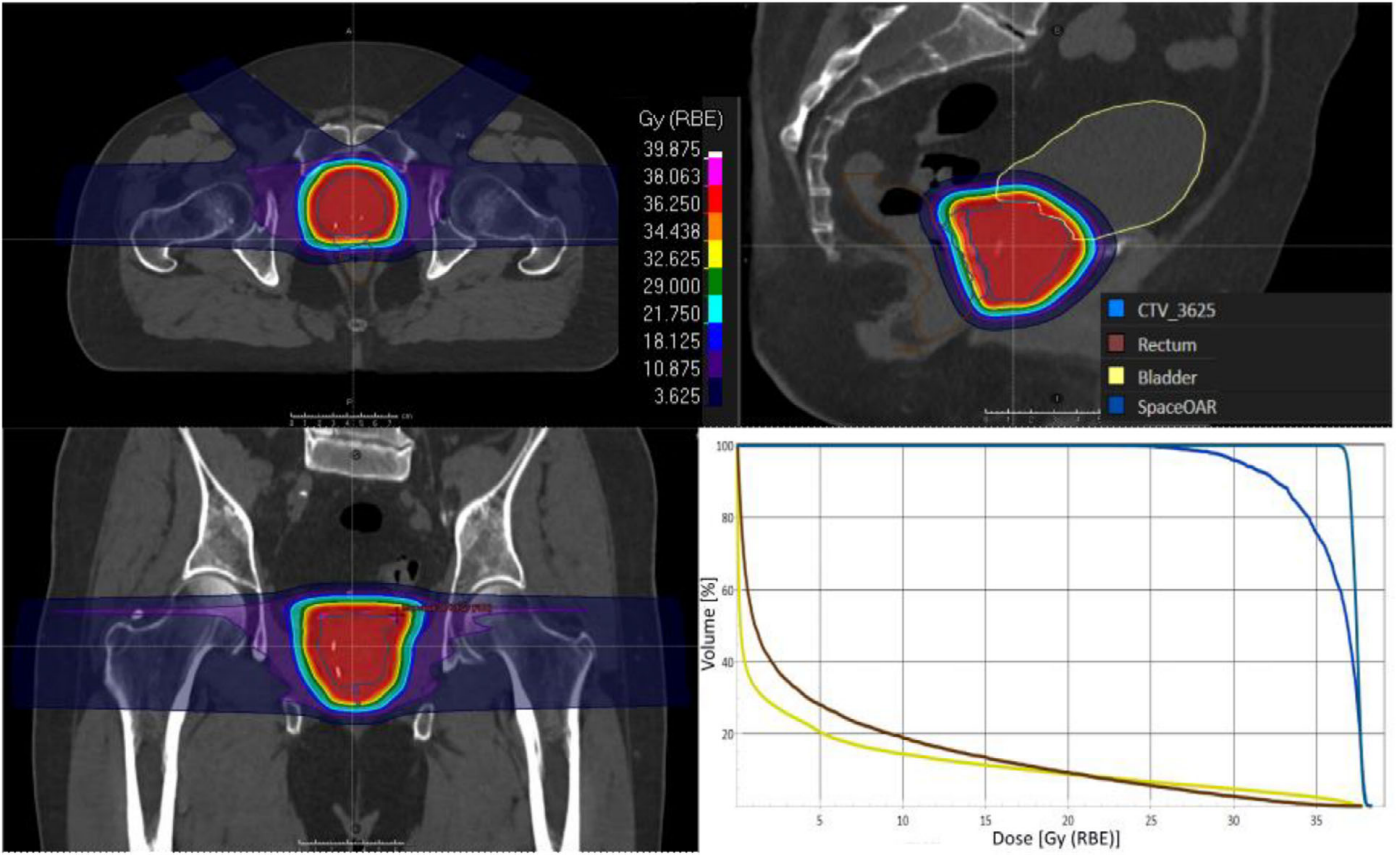
Axial, Coronal, and Sagittal views of dose distribution and the dose volume histogram of a prostate SBRT plan on the initial planning CT showing the target and organs at risk. SBRT, stereotactic body radio therapy.

All patients were planned with two lateral beams, contributing 70% of the prescribed dose, and two anterior oblique beams, contributing 30%, as shown in Figure 1. These beam angles were chosen to optimize dose conformity to the CTV while minimizing dose to the bladder, rectum, and femoral heads. The anterior oblique beams helped reduce the impact of interfractional shift of the prostate relative to the femur bones, while the lateral beams allowed for dose delivery to the entire prostate with reduced impact on the bladder and rectum. Robust optimization, incorporating a 5 mm setup margin in all orthogonal directions except posterior (3 mm) and a 3.5% range uncertainty, was essential to account for setup uncertainties and interfractional anatomical variations, such as bladder filling and prostate motion, ensuring consistent CTV coverage despite daily anatomical changes.

The initial treatment plan was applied to Hounsfield Unit (HU) and artifact‐corrected pre‐treatment cone‐beam CT (CBCT) images to calculate the delivered dose for each fraction.^30^

### 2.3 Treatment delivery

Patients were positioned using fiducial‐based alignment, followed by CBCT to verify the prostate's position relative to surrounding anatomy, bladder and rectum filling, and body surface at the beam entrance. Orthogonal kV imaging was used to further verify fiducial positions before beam delivery. Pre‐treatment CBCTs and orthogonal kV images were performed at each fraction to ensure anatomical consistency with the planning CT. Bladder and rectal filling were assessed on CBCT, with adjustments (e.g., additional water intake or partial release) made as needed.

### 2.4 Dosimetric and anatomical data extraction

Since CBCTs are aligned to the treatment plan CT (TPCT) based on fiducial markers, the absolute prostate position shift compared to the TPCT is not an ideal representation of inter‐fraction prostate motion. In this study, prostate shifts relative to the femoral heads were used instead. The distance from the center of the prostate to the average of the left/right femoral heads’ most superior/anterior coordinates of their bounding boxes was calculated to represent the prostate's superior‐inferior (SI) and anterior‐posterior (AP) positions, respectively, as described in Chang et al.^31^ Specifically, the prostate center coordinates (P(x_c_, y_c_, z_c_)) were compared to the superior (*z*‐direction) and anterior (*y*‐direction) edges of the femoral head bounding boxes:

(1) $$\mathrm{Prostate}\;\mathrm{SI}\;\mathrm{position}\;=\left|z_{c}-\frac{z_{1}+z_{2}}{2}\right|$$

(2) $$\mathrm{Prostate}\;\mathrm{AP}\;\mathrm{position}\;=\left|y_{c}-\frac{y_{1}+y_{2}}{2}\right|$$

**FIGURE 2:**
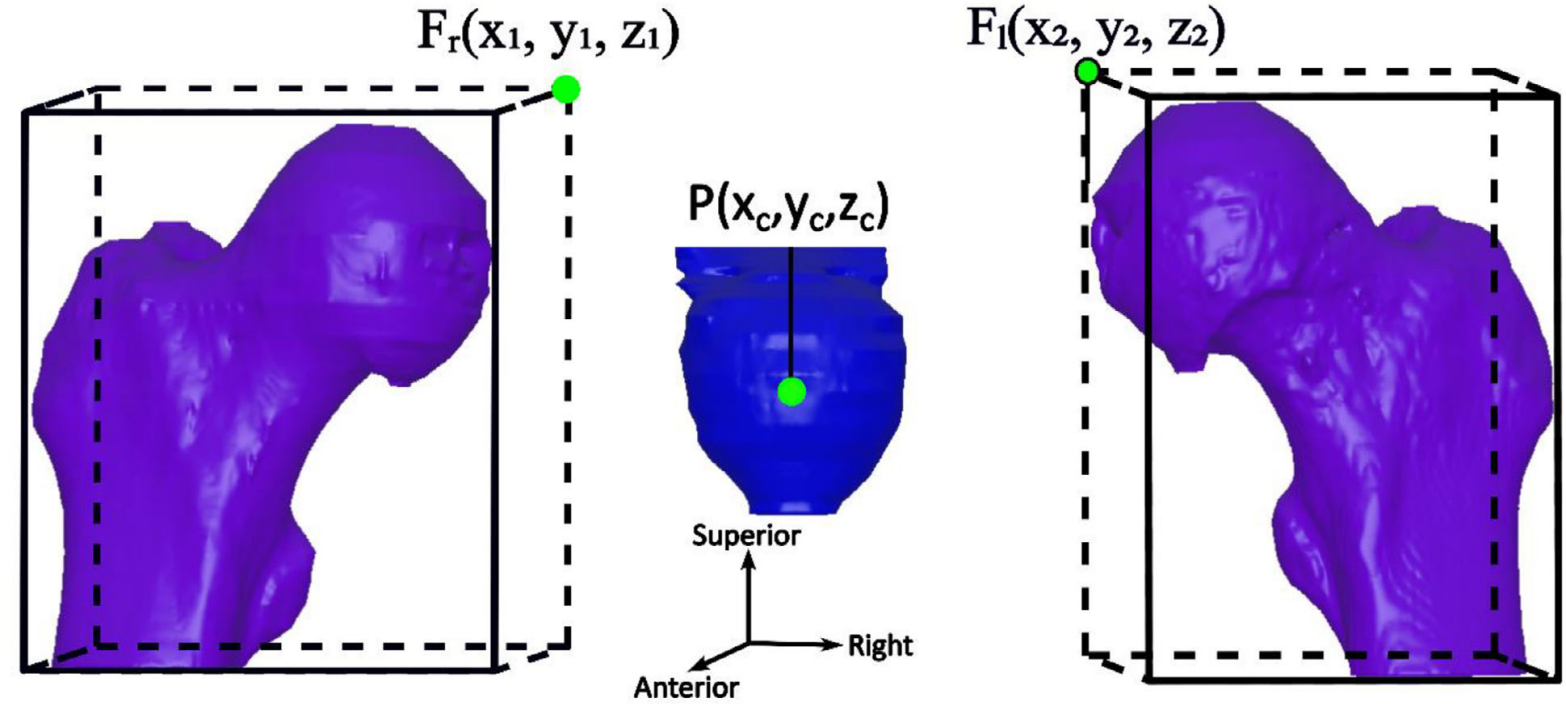
Schematic of prostate shift calculation relative to bounding boxes of femoral heads, showing AP/SI axes and reference landmarks used in the analysis. The anterior/superior coordinates of the right femoral (F_r_) and left femoral (F_l_) head bounding boxes were averaged and compared to the prostate center (P) to compute daily relative positions and interfractional shifts. AP, anterior‐posterior; SI, Superior‐Inferior.

To quantify daily shifts due to anatomical variation, the interfractional shift in each direction was calculated as the difference between the prostate's relative position on the daily CBCT and its planned position at simulation. A schematic of this calculation is provided in Figure 2, showing the spatial relationship between the prostate center and bilateral femoral head landmarks used for shift quantification.

Key dosimetric parameters analyzed included the dose delivered to 99% of the clinical target volume (CTV D_99_) and the maximum dose to the CTV (CTV D_0.03cc_). For the OARs, maximum doses to the rectum (rectum D_0.03cc_) and bladder (bladder D_0.03cc_) were evaluated. Anatomical metrics included bladder and rectum volumes, along with SI and AP shifts in the CTV. Lateral (R/L) shifts were not considered, as the prostate exhibits minimal motion in this direction due to pelvic bony constraints. Additionally, proton PBS delivery is more sensitive to range uncertainties and anatomical variations in the beam path (typically AP or lateral beams), making SI/AP shifts more relevant to dosimetric variations in this setting.

A custom RayStation script facilitated the extraction of these metrics for each fraction, enabling a detailed comparison of planned versus delivered doses. A total of 105 fractions were analyzed across the 42 patients. The delivered dose was obtained by applying the original treatment plan to the HU‐ and artifact‐corrected pre‐treatment CBCT (cCBCT) images. Deformable image registration (DIR) was then used to map the fraction doses onto the planning CT, allowing for cumulative dose evaluation. This approach accounted for daily anatomical variations without introducing additional uncertainties from shifting the planning CT. Each of these metrics was obtained for both the planned and delivered doses to enable a detailed comparison of dose deviations resulting from interfractional variations.

### 2.5 Statistical analysis

Anatomical changes, including bladder and rectum volumes and shifts in the CTV (SI and AP directions), were analyzed to evaluate their impact on dosimetric changes. The dosimetric metrics were chosen based on their clinical relevance in treatment planning and predicting toxicity outcomes:

1. CTV D_99_: Minimum dose delivered to 99% of the CTV, reflecting target coverage.
2. CTV D_0.03cc_: Maximum dose delivered to the CTV, indicating hotspots.
3. Bladder D_0.03cc_: Maximum dose to the bladder, a predictor of genitourinary toxicity.
4. Rectum D_0.03cc_: Maximum dose to the rectum, a factor associated with rectal toxicity.

Dosimetric changes were binarized using treatment planning objectives: CTV D_99_ (< 36.3 Gy or 39 Gy), CTV D_0.03cc_ (> 110% of the prescription dose), bladder D_0.03cc_ (> 37.8 Gy or 41.5 Gy), and rectum D_0.03cc_ (> 37.5 Gy). These thresholds were chosen to identify under‐coverage, hotspots, or excessive dose delivery to critical structures. By concentrating on these key parameters, we aimed to provide a focused and meaningful analysis of the dosimetric impact of interfractional variations.

**FIGURE 3:**
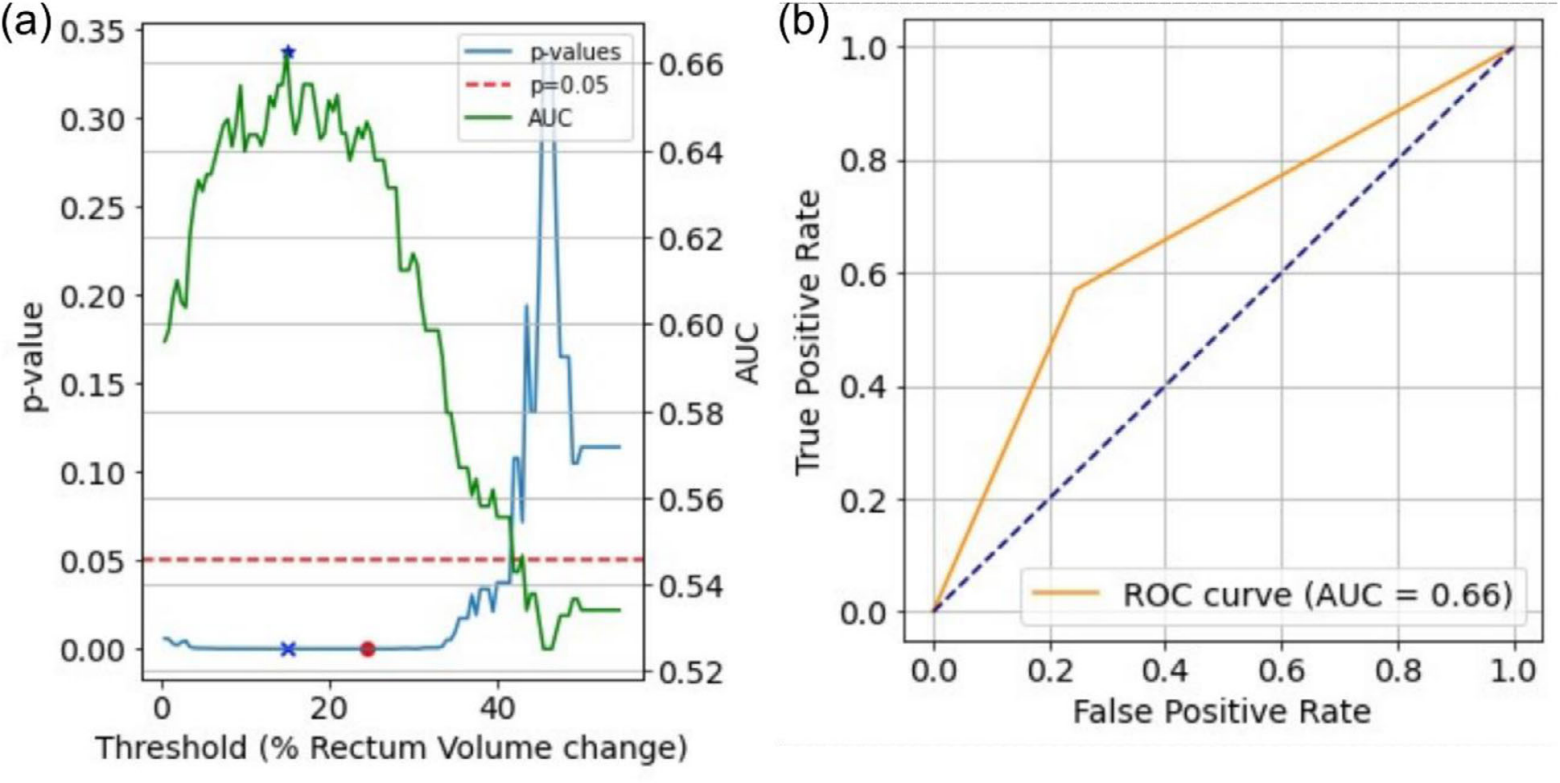
(a) *p*‐values and AUC as a function of rectum volume change thresholds predicting rectum D_0.03cc_ > 37.5 Gy. The *p*‐values are displayed alongside AUC values for thresholds from 0 to 54.5%, with significance indicated at *p* = 0.05. Red dot indicates the lowest *p*‐value, blue x denotes the *p*‐value at the selected threshold (15%), and blue * indicates the AUC at the selected threshold. (b) ROC curve showing the diagnostic performance of the selected rectum volume threshold model. The model achieves an AUC of 0.66, indicating moderate predictive ability. AUC, area under the curve.

Thresholds for these anatomical changes were incrementally increased by 0.5%, and logistic regression models were applied at each threshold to predict corresponding dosimetric changes. Chi‐square tests calculated *p*‐values for each threshold, and Area Under the Curve (AUC) quantified the model's discriminative power (Figure 3a,b). The threshold with the highest AUC and a *p*‐value < 0.05 was selected as the optimal threshold for predicting significant dosimetric changes. To account for multiple comparisons, a Benjamini–Hochberg false discovery rate (FDR) correction was applied to the *p*‐values obtained from chi‐square tests, reducing the risk of false positives while maintaining statistical power.

Initially, univariate logistic regression models were applied to assess the individual impact of each anatomical factor on dosimetric deviations. Significant predictors from the univariate analysis (*p* < 0.05) were then incorporated into multivariate logistic regression models to evaluate the combined effects of multiple anatomical factors. To further explore relationships between anatomical variations and dosimetric outcomes, multivariate logistic regression models were fit to assess the combined effect of multiple anatomical factors. Odds ratios (OR) and their corresponding 95% confidence intervals (CIs) were computed for each anatomical factor. AUC values were also calculated to evaluate the overall predictive accuracy of each model, with *p*‐values < 0.05 considered statistically significant for all analyses. All statistical analyses were conducted using Python (version 3.9.11) and SciPy (version 1.8.1).

## 3 RESULTS

### 3.1 Patient characteristics and general dosimetric results

**FIGURE 4:**
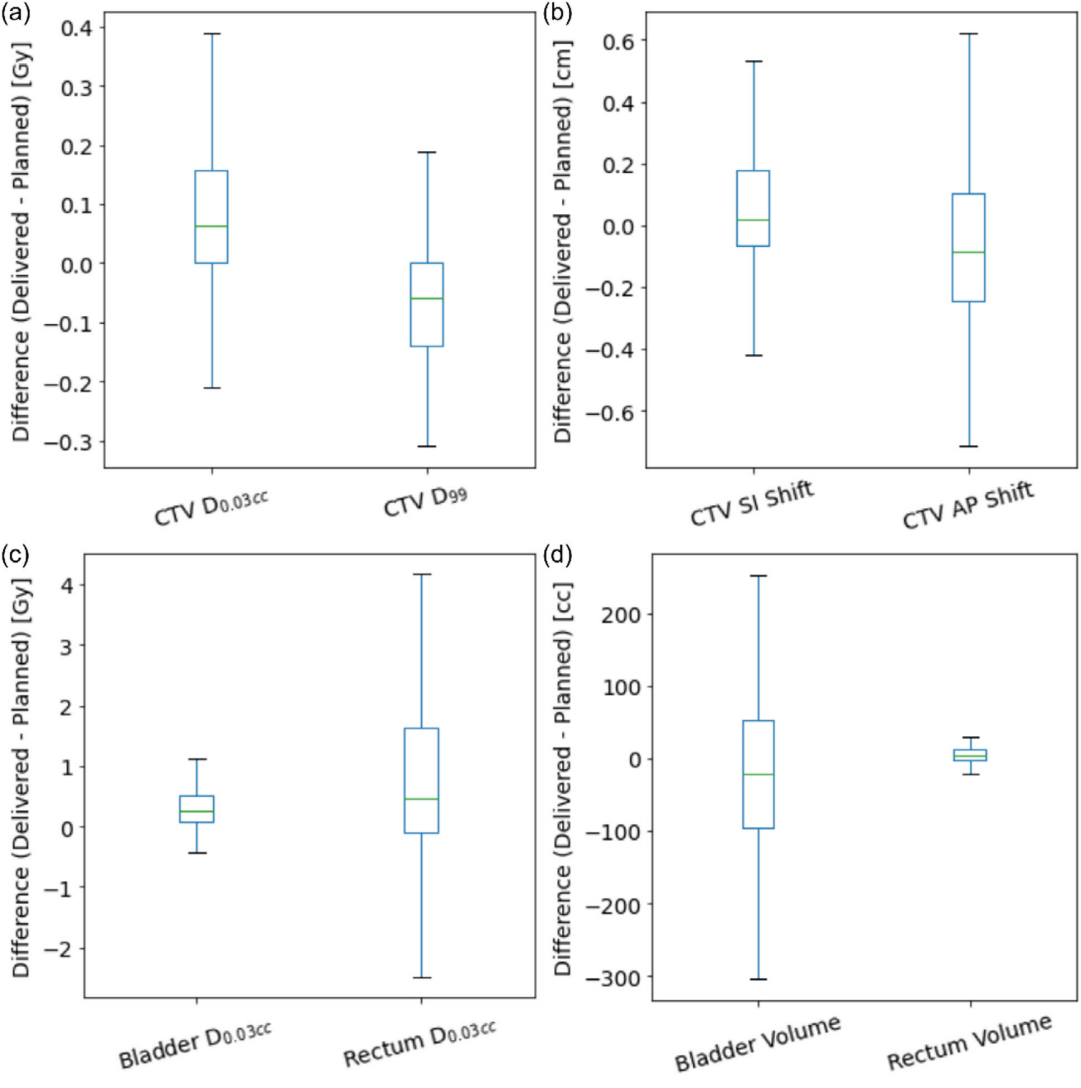
Difference between the delivered and planned (a) CTV Doses, (b) CTV Shifts, (c) bladder and rectum D_0.03cc,_ and (d) bladder and rectum volumes. CTV, clinical target volume.

A total of 42 patients with localized PCa were treated with proton PBS‐SBRT during the selected period. The delivered CTV coverage was slightly lower than planned for CTV D_99_: −0.28% ± 1.00% (*p* < 0.001) and slightly higher for CTV D_0.03cc_: 0.26% ± 0.61% (*p* < 0.001). For OARs, the bladder D_0.03cc_ and rectum D_0.03cc_ showed higher delivered doses than planned, with mean deviations of 0.33 ± 0.38 Gy, and 0.68 ± 2.48 Gy, respectively. Table 1 summarizes the planned and delivered dosimetric and anatomical properties of the cohort. Figure 4 illustrates the deviations between planned and delivered properties, including dose metrics, CTV shifts, and bladder and rectum volumes.

**TABLE 1:** Summary of planned and delivered dosimetric and anatomical parameters for the 42 patients treated with PBS‐SBRT for localized PCa.

Dosimetric/Anatomical Parameters	Planned (Median, Range)	Delivered (Median, Range)
Prescription dose (Gy)	36.25 (*n* = 41), 40 (*n* = 1)	36.25 (*n* = 41), 40 (*n* = 1)
SI shift in CTV (cm)	−3.77 (−5.82, −1.92)	−3.73 (−6.19, −1.86)
AP shift in CTV (cm)	−3.79 (−5.76, −0.74)	−3.84 (−5.73, −0.74)
Bladder volume (cc)	277.19 (71.19, 716.47)	276.07 (54.27, 1035.79)
Rectum volume (cc)	57.67 (33.08, 98.44)	63.15 (32.91, 186.06)
CTV D_99_ (Gy)	36.45 (36.02, 38.22)	36.36 (31.92, 39.09)
CTV D_0.03cc_ (Gy)	38.03 (37.46, 41.90)	38.32 (37.46, 42.85)
Bladder D_0.03cc_ (Gy)	37.74 (37.00, 41.44)	38.03 (36.83, 42.00)
Rectum D_0.03cc_ (Gy)	36.50 (25.07, 41.32)	37.07 (22.59, 42.01)

*Note*: Continuous values are reported as median (range) for both patient characteristics and dosimetric metrics. Planned values for SI and AP shifts correspond to the initial setup positions at simulation, while delivered values represent the actual interfractional variations measured relative to the femoral heads at treatment fractions.

Abbreviations: PCa, prostate cancer; PBS, pencil beam scanning; SBRT, stereotactic body radiotherapy.

### 3.2 Univariate analysis

Univariate analysis identified anatomical variations significantly associated with dosimetric deviations. For CTV D_99_, bladder volume increases of more than 57.5% (177.85 cc) were the strongest predictor, with an OR of 0.30, AUC of 0.57, and *p* = 0.03. For CTV D_0.03cc_, an AP shift threshold of 7.5% (−0.27 cm) showed the strongest association, with an OR of 0.35, AUC of 0.59, and *p* = 0.01 (Table 2). Rectum volume increases were significantly associated with rectum D_0.03cc_ deviations (OR = 4.09, AUC = 0.66, *p* = 0.01). Full results are detailed in Table 2.

**TABLE 2:** Summary of chi‐square, *p*‐values, OR, and AUC for the association between dosimetric feature changes and anatomical feature changes at various thresholds.

Dosimetric feature change	Anatomical feature change (Threshold) (mean)	Chi‐square	*p*‐value	OR	AUC
CTV D_99_ < 36.3 Gy or 39 Gy	AP Shift in CTV (15%) (−0.54 cm)	1.98	0.243		–
	SI shift in CTV (4%) (−0.15 cm)	3.57	0.122		–
	**Bladder volume (57.5%)** **(177.85 cc)**	**7.11**	**0.032**	**0.30**	**0.57**
	Rectum volume (20%) (12.29 cc)	1.42	0.288		–
CTV D_0.03cc_ > 110% Rx	**AP shift in CTV (7.5%)** (−**0.27 cm)**	**6.80**	**0.011**	**0.35**	**0.59**
	SI shift in CTV (7%) (‐0.26 cm)	1.76	0.245		–
	**Bladder volume (35.5%)** **(109.80 cc)**	**7.92**	**0.032**	**4.69**	**0.58**
	Rectum volume (3%) (1.84 cc)	2.09	0.237		–
Bladder D_0.03cc_ > 37.8 Gy or 41.5 Gy	AP shift in CTV (6.5%) (‐0.24 cm)	1.15	0.313		–
	SI shift in CTV (6.5%) (‐0.25 cm)	2.45	0.199		–
	Bladder volume (7%) (21.65 cc)	1.62	0.260		–
	Rectum volume (21%) (12.91 cc)	0.80	0.384		–
Rectum D_0.03cc_ > 37.5 Gy	AP shift in CTV (8%) (−0.29 cm)	5.24	0.07		–
	SI shift in CTV (8.5%) (‐0.32 cm)	2.50	0.120		–
	Bladder volume (8%) (24.74 cc)	3.98	0.107		–
	**Rectum volume (15%)** **(9.22 cc)**	**21.08**	**0.011**	**4.09**	**0.66**

Note: The table shows significant anatomical changes, such as AP and SI shift in CTV, bladder volume, and rectal volume, correlated with changes in CTV D_99_, CTV D_0.03cc_, Bladder D_0.03cc_, and Rectum D_0.03cc_. Significant results (*p* < 0.05) are indicated in bold.

Abbreviations: AP, anterior‐posterior; AUC, area under the curve; CTV, clinical target volume; ORs, odds ratios; SI, superior‐inferior.

### 3.3 Multivariate analysis

**FIGURE 5:**
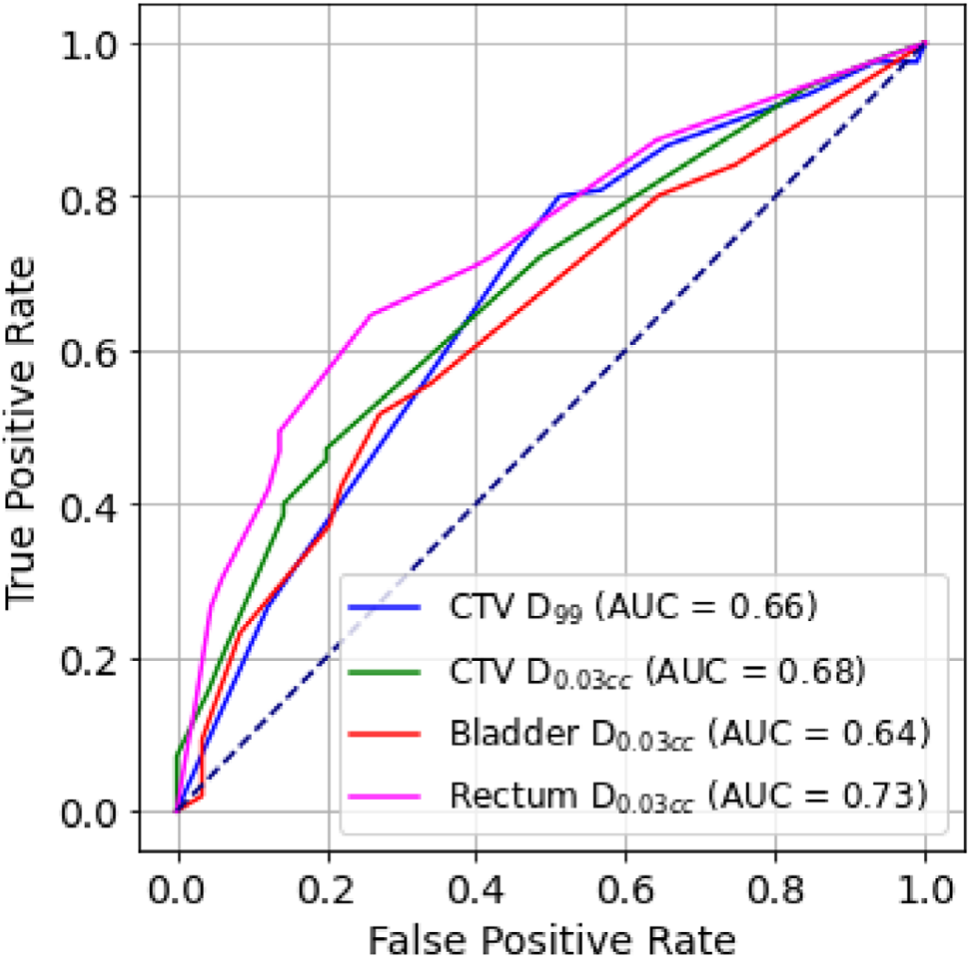
ROC curves showing the performance of multivariate logistic regression models for predicting different dosimetric outcomes: CTV D_99_ (< 36.3 or 39 Gy), CTV D_0.03cc_ (> 110% prescription dose), bladder D_0.03cc_ (> 37.8 Gy or 41.5 Gy), and rectum D_0.03cc_ (> 37.5 Gy). These dosimetric outcomes were binarized based on clinically relevant thresholds. CTV D_0.03cc_ demonstrated the highest AUC (0.73), followed by rectum D_0.03cc_ (0.73), bladder D_0.03cc_ (0.68), and CTV D_99_ (0.63). The models included anatomical feature changes such as shifts in the CTV and changes in bladder and rectum volumes. AUC, area under the curve; CTV, clinical target volume.

**FIGURE 6:**
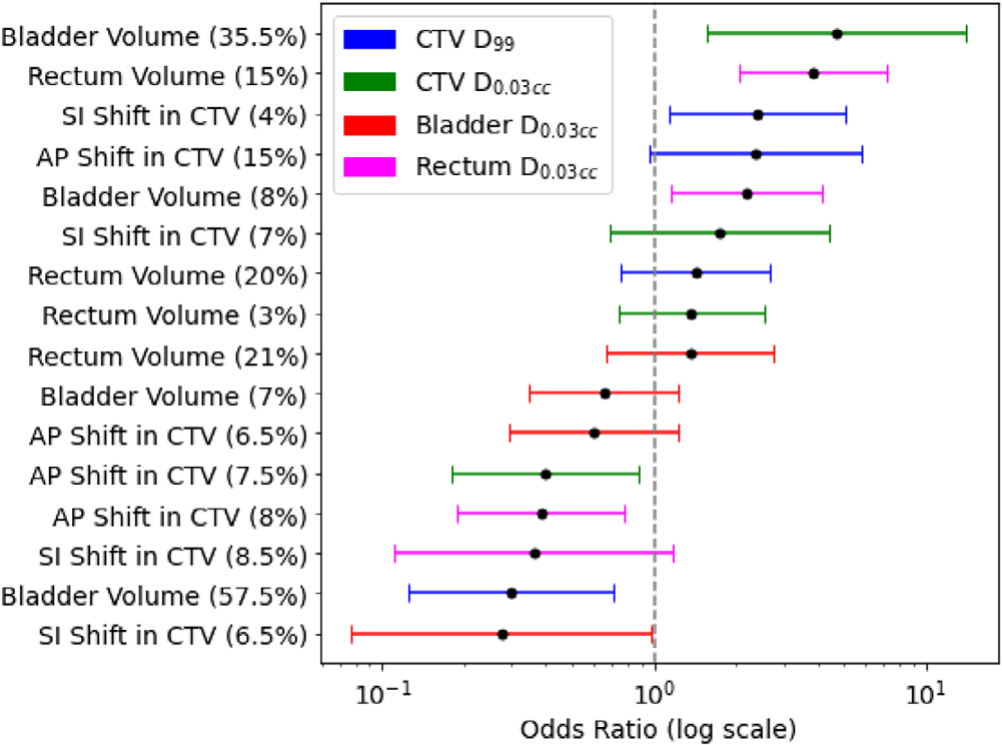
Forest plot showing ORs and 95% CIs for anatomical feature changes predicting various dosimetric outcomes: CTV D_99_ (< 36.3 or 39 Gy), CTV D_0.03cc_ (> 110% of the prescription dose), bladder D_0.03cc_ (> 37.8 Gy or 41.5 Gy), and rectum D_0.03cc_ (> 37.5 Gy). The anatomical features include bladder volume, rectum volume, and shifts in the CTV (AP and SI), with percentage increases shown in parentheses. ORs greater than 1 suggest an increased likelihood of the corresponding dosimetric feature change due to anatomical variations. CI that do not cross the vertical line at OR = 1 are considered statistically significant. AP, anterior‐posterior; CI, Confidence intervals; CTV, clinical target volume; ORs, odds ratios; SI, Superior‐inferior.

Multivariate logistic regression models demonstrated improved predictive accuracy when combining multiple anatomical factors. For CTV D_0.03cc_, a model incorporating AP and SI shifts alongside bladder and rectum volumes achieved an AUC of 0.73. Similarly, the model for rectum D_0.03cc_ also achieved an AUC of 0.73, highlighting the importance of anatomical variations. Bladder volume (OR = 4.70, *p* = 0.03) was the most significant predictor of CTV D_0.03cc_ deviations. For rectum D_0.03cc_, rectum volume increases above 15% (OR = 3.83, *p* = 0.01) were the strongest predictors, as shown in Table 3 and Figures 5 and 6. These findings suggest that anatomical monitoring and adaptive planning strategies can mitigate the impact of interfractional variations.

**TABLE 3:** ORs, *p*‐values, and AUC for the association between dosimetric feature changes and anatomical feature changes at specific thresholds.

Dosimetric feature change	Anatomical feature change (Threshold)	OR	*p*‐value	AUC
CTV D_99_ < 36.3 Gy or 39 Gy	AP shift in CTV (15%)	2.36	0.122	0.66
	SI shift in CTV (4%)	2.40	0.107	
	**Bladder volume (57.5%)**	**0.30**	**0.032**	
	Rectal volume (20%)	1.42	0.313	
CTV D_0.03cc_ > 110% Rx	AP shift in CTV (7.5%)	0.40	0.069	0.68
	SI shift in CTV (7%)	1.73	0.292	
	**Bladder volume (35.5%)**	**4.70**	**0.032**	
	Rectal volume (3%)	1.37	0.332	
Bladder D_0.03cc_ > 37.8 Gy Or 41.5 Gy	AP shift in CTV (6.5%)	0.60	0.243	0.64
	SI shift in CTV (6.5%)	0.28	0.107	
	Bladder volume (7%)	0.65	0.245	
	Rectal volume (21%)	1.36	0.399	
Rectum D_0.03cc_ > 37.5 Gy	**AP shift in CTV (8%)**	**0.38**	**0.032**	0.73
	SI shift in CTV (8.5%)	0.36	0.171	
	**Bladder volume (8%)**	**2.20**	**0.050**	
	**Rectal volume (15%)**	**3.83**	**0.011**	

*Note*: The table highlights the anatomical changes significantly associated with CTV D99, CTV D_0.03cc_, bladder D_0.03cc_, and rectum D_0.03cc_. Statistically significant results (*p* < 0.05) and their corresponding ORs are shown, with AUC values indicating the predictive performance of each model.

Abbreviation: AP, anterior‐posterior; AUC, area under the curve; CTV, clinical target volume; ORs, odds ratios; SI, superior‐inferior.

## 4 DISCUSSION

This study investigated the dosimetric impact of interfractional anatomical variations during PBS‐SBRT for localized PCa. Our findings reveal significant associations between anatomical changes and deviations in key dosimetric parameters, emphasizing the importance of adaptive planning to mitigate these effects.

For CTV D_99_ and CTV D_0.03cc_, AP shifts in the CTV were significantly predicted dose deviations. AP shifts exceeding 7.5% (mean: −0.27 cm) significantly influenced CTV D_0.03cc_ with an OR of 0.35 (*p* = 0.01), and an AUC of 0.59. This underscores the high sensitivity of dosimetric accuracy to even minor anatomical variations, reinforcing the need for precise daily monitoring, especially in treatments with tight margins like SBRT.^32^, ^33^

Bladder and rectum volumes also demonstrated strong associations with dosimetric deviations. Bladder volume increases of 35.5% (109.80 cc) were significantly linked to deviations in CTV D_0.03cc_, with an OR of 4.69 and an AUC of 0.58. Similarly, rectum volume changes exceeding 15% (9.22 cc) were predictive of elevated rectum D_0.03cc_, with an OR of 4.09 (*p* = 0.01), and the highest univariate AUC of 0.66 among all metrics. Our findings are consistent with previous studies that have highlighted the role of interfractional changes in influencing dosimetric outcomes during proton therapy for PCa. Moteabbed et al. showed that while both proton and photon therapies provided acceptable target coverage, interfractional anatomical changes significantly impacted bladder and rectal doses in both modalities.^34^ Similarly, Wang et al. reported that interfractional variations, particularly rectal gas volume and bladder filling, led to dose deviations from the simulation plan during proton therapy, findings that align closely with our results.^35^ These findings emphasize the critical need to manage bladder and rectum volumes during PBS‐SBRT, as even moderate changes can result in significant dosimetric deviations. Such management is essential for maintaining target coverage and sparing OARs, especially given the high doses used in SBRT.

The results demonstrate the value of combining anatomical changes to better understand dosimetric deviations. While individual anatomical factors provide key insights, multivariate analysis highlights their combined effects in capturing the complexity of interfractional variations, allowing for more accurate predictions. For example:

1. Rectum D_0.03cc_: Including bladder and rectum volumes along with AP shifts increased the AUC from 0.66 (univariate) to 0.73 (multivariate), underscoring the added value of combining predictors.
2. CTV D_0.03cc_: Multivariate analysis revealed a synergistic effect between AP shifts and bladder volumes, yielding an AUC of 0.68 compared to lower values in univariate models.

This improvement highlights the ability of multivariate models to account for the combined effects of anatomical changes, capturing the complexity of interfractional variations more effectively than univariate analyses.

Figure 6 illustrates the ORs and CIs for the key predictors in multivariate models. Notably, bladder volume changes exceeding 35.5% were associated with a five‐fold increased likelihood of CTV D_0.03cc_ deviations (OR = 5.78, *p* = 0.03). An OR of 5.78 indicates that patients with bladder volume increases above 35.5% are approximately five times more likely to experience deviations in CTV D_0.03cc_ compared to those with smaller changes. Rectum volume changes of 15% were the strongest predictors of rectum D_0.03cc_ deviations (OR = 4.23, *p* = 0.01). If a CI does not include OR = 1, it means the predictor is statistically significant and strongly linked to dosimetric deviations. For example, bladder volume change for CTV D_99_ and rectum volume change for rectum D_0.03cc_ show strong relationships with dosimetric deviations. ORs > 1 suggest a positive association (increased likelihood of dosimetric deviations), while ORs < 1 indicate a negative association (reduced likelihood).

Although the multivariate models provide useful predictions, the AUC values suggest that additional factors beyond anatomical changes contribute to dosimetric deviations. Notably, we observed that CTV D_99_ tended to decrease with anatomical shifts, indicating potential underdosing of the target, whereas rectum and bladder D_0.03cc_ values generally increased, suggesting higher OAR exposure. Intra‐fractional motion, tissue heterogeneity, and setup uncertainties are likely contributors. Future studies should explore additional sources of variability, such as inter‐observer contouring differences, to better explain the remaining dose discrepancies and refine adaptive strategies. Feng et al. developed a novel 4D voxel‐based method to assess intra‐fraction prostate motion in hypofractionated proton SBRT.^36^ Their findings demonstrated that, even in cases with notable prostate motion, the dose distribution remained largely unaffected in 95% of cases. This underscores the importance of motion management in ensuring dose accuracy, particularly in the context of ultrahypofractionation where high doses per fraction are used. The integration of real‐time motion tracking, as demonstrated by Feng et al, is crucial in mitigating dosimetric uncertainties during proton SBRT and maintaining target coverage despite anatomical shifts.

cCBCT has been proved to be a valuable tool in managing interfractional variations observed in this study by refining CBCT intensity values to align with planning CT for improved daily dose monitoring.^30^ Despite its potential, cCBCT faces challenges such as residual artifacts and limited field‐of‐view (FOV), which can hinder accurate visualization of key structures like the bladder, adding uncertainty to dose delivery.

AI‐based techniques for generating synthetic CT images from CBCT scans have shown promise in mitigating artifacts and improving dose calculations.^37^, ^38^ These methods could enhance adaptive workflows for PBS‐SBRT by providing CT‐like images with accurate HU values, potentially enabling precise daily dose adjustments.^39^ However, these methods require validation across diverse clinical cohorts to ensure reliability and integration into routine workflows, necessitating improvements in computational efficiency and regulatory alignment.

The results of this study have direct implications for clinical practice, particularly in the context of adaptive radiotherapy. The identification of significant anatomical predictors, such as bladder and rectum volumes, and CTV shifts relative to the femur, suggests that daily monitoring of these parameters could help optimize treatment delivery. Adaptive planning strategies that account for daily anatomical changes could reduce the risk of target underdosing and OAR overdosing, thereby improving treatment outcomes.

Future studies should prioritize understanding and addressing the dosimetric impact of complex anatomical changes, such as prostate deformation, bladder filling, and rectal distension, which can influence dose distribution during PBS‐SBRT. Investigating strategies to integrate real‐time motion tracking and adaptive radiotherapy protocols may further enhance treatment precision, minimizing deviations and improving outcomes. Additionally, advancing imaging technologies and exploring novel approaches, such as AI‐based synthetic CT generation, will be critical for enabling more robust adaptive workflows in clinical practice. These efforts should aim to balance computational efficiency with clinical feasibility, ensuring seamless integration into routine care while maintaining patient safety and treatment accuracy.

## 5 CONCLUSION

This study underscores the critical impact of interfractional anatomical variations on dosimetric deviations during PBS‐SBRT for localized PCa. Significant predictors, including CTV shifts relative to femur bony anatomy (AP/SI directions) and bladder and rectal volume changes, highlight the necessity of precise daily anatomical monitoring to mitigate dose deviations and optimize treatment outcomes. Emerging technologies, such as corrected CBCT and AI‐generated synthetic CT, hold substantial potential for improving real‐time dose adaptation. However, challenges remain in validating these tools, ensuring their clinical integration, and addressing computational and workflow barriers. Future efforts should prioritize refining these imaging modalities and advancing adaptive radiotherapy workflows to enhance treatment precision, reduce toxicity risks, and improve patient outcomes.

## AUTHOR CONTRIBUTION

Keyur D. Shah: Data curation; Software; Formal analysis; Investigation; Writing—original draft; Visualization. Duncan H. Bohannon: Data curation; Writing—review & editing. Sagar A. Patel: Writing—review & editing. Chih‐Wei Chang: Writing—review & editing. Vishal R. Dhere: Writing—review & editing. Yinan Wang: Writing—review & editing. Anees Dhabaan: Writing—review & editing. Hania Al‐Hallaq: Writing—review & editing. Xiaofeng Yang: Writing—review & editing. Jun Zhou: Conceptualization; Methodology; Supervision; Project administration; Writing—review & editing.

## CONFLICT OF INTEREST STATEMENT

The authors declare no conflicts of interest.
